# Corrected Axial Length and Choroidal Thickness: A Correlation Analysis for Scientific Purposes

**DOI:** 10.3390/jpm15010015

**Published:** 2025-01-02

**Authors:** Marco Gioia, Maddalena De Bernardo, Ferdinando Cione, Martina De Luca, Nicola Rosa

**Affiliations:** 1 Eye Unit—Department of Medicine, Surgery, Dentistry “Scuola Medica Salernitana”, University of Salerno, via S. Allende, 84081 Baronissi, Italy; mgioia@unisa.it (M.G.); mdebernardo@unisa.it (M.D.B.); nrosa@unisa.it (N.R.); 2 AOU San Giovanni di Dio e Ruggi D’Aragona, 84131 Salerno, Italy

**Keywords:** axial length, choroidal thickness, corrected axial length, ChT-AL correlation

## Abstract

**Purpose:** Choroidal thickness (ChT) is an important measurement for evaluating eye and systemic disorders, but it is influenced by numerous elements, especially axial length (AL). It is known that the presence of a linear relationship between ChT and AL exists, but recently it has been shown that the AL measurement obtained with the current optical biometry is not very precise and needs to be corrected. This study aimed to verify if a similar correlation also persists with this corrected AL (ALc). **Methods:** All subjects underwent a complete eye examination, including spectral domain optical coherence tomography (OCT) with enhanced depth image (EDI) mode and AL measurement with IOLMaster. After a normality check of the data, the correlations between ChT with AL and ALc were investigated through the Pearson correlation coefficient. *p* values < 0.05 were considered statistically significant. **Results:** In total, 100 eyes of 50 healthy patients were evaluated. The mean AL was 24.36 ± 1.23 mm and mean ALc was 24.25 ± 1.22 mm. The mean nasal ChT, subfoveal ChT, and temporal ChT were, respectively, 250.57 ± 93.93 µm, 307.18 ± 101.66 µm, and 313.72 ± 88.86 µm. A significant negative linear correlation was found by comparing both AL and ALc to ChT (all r < −0.500, all *p* < 0.050). The negative linear correlation was stronger between nasal ChT and both AL and ALc (all r = −0.581). **Conclusions:** Through OCT and optical biometry, we confirmed that a statistically significant correlation persists between ALc and ChT, equal to the uncorrected AL. On these bases, in ChT studies or protocols, we recommend stratifying population according to ALc because linear correlation is still present; however, the cut-off values should be changed according to the systematic errors in optical biometry. In addition, both AL and ChT changes should be evaluated according to ALc.

## 1. Introduction

Choroid evaluation is becoming an emerging topic in ophthalmology due to its role in the diagnosis and follow-up of several pathologies and conditions [1,2,3]. Spaide et al., with their pioneering studies, first described a method of obtaining images of the choroid using conventional optical coherence tomography (OCT) with the aim of evaluating choroidal thickness (ChT) measurements using these images [4,5,6]. In fact, in the last few years, several authors investigated the role of ChT in several systemic and eye diseases, even though the assessment of this choroidal parameter is very difficult because it is affected by several factors, such as body position, axial length (AL), drug intake, and drug withdrawal [1,2]. The relationship between AL and ChT has been studied in detail: in fact, it is known in the scientific literature that a significant relationship exists between AL and ChT [2,3,7,8,9,10,11,12,13,14,15,16,17]. A linear relationship seems to be present between AL and ChT, with short eyes having a thicker choroid, and long eyes having a thinner one [3].

For this reason, in a study evaluating ChT and ChT changes, it essential to stratify subjects according to AL, in order to obtain homogeneous and comparable samples. However, studies that evaluate ChT generally do not consider that AL evaluation, especially in patients affected by cataracts, are more challenging and the risk of bias is really high. In fact, especially in cases of biometry being based on group refractive index (GRI) or when evaluating extremely long eyes, AL measurements need to be corrected [18].

Several authors proposed different AL correcting factors [18,19,20,21,22], but most of them are strictly designed for IOL power calculation [19,20,21,22]. Instead, other correcting equations are based on erroneous AL measurements due to lens opacity or GRI-based biometry [18,22], such as the corrected AL (ALc) by De Bernardo et al. [18].

Corrected ALs potentially have a double impact in ChT evaluation studies:

(1) A change in the relationship between AL and ChT measurements could occur;
(2) More important, the choice of AL cut-off values in these types of studies could change accordingly.

Regarding point 1, if we consider that many AL correction factors are generally applied only in specific AL ranges [19,20,21,22], they could influence the linear relationship with a “zone of conflict” when passing the cut-off values for adjustment application. For example, Wang–Koch adjustments, which are usually applied in case of long eyes, could modify this linear relationship.

Regarding point 2, the cut-off values of AL in ChT studies become crucial: the choice of the right values is essential for patients’ recruitment, samples stratifications, change analysis, and outcome evaluations. To better understand the impact of corrected AL on ChT studies, some examples of AL/ChT studies are reported:

- AL changes related to ChT changes in pharmacological studies [23];
- AL and ChT changes in myopia studies in children and adolescents [24];
- AL and ChT changes in myopia studies in adults [25];
- AL and ChT changes in accommodation studies [14];
- AL stratification in ChT studies [26].

Why do we pay great attention to ChT reliability, and also forget to take AL reliability in choroidal studies into consideration?

We tried to answer this question and so far, to the best of our knowledge, no studies investigated the influence of corrected AL with ChT. Does the relationship between AL and ChT remain unaltered? If not, how should studies evaluating ChT stratify samples? The purpose of this study was to evaluate the correlation between ChT and ALc, because only this latter AL adjustment is not exclusively linked to IOL power calculation accuracy; however, it eliminates systematic error in biometry.

## 2. Materials and Methods

### 2.1. Participants

In this study, healthy subjects with no ocular diseases which could influence the AL or OCT measurement, such as cataract or corneal and vitreous opacities, were recruited between September 2022 and January 2023. The exclusion criteria were systemic and ocular diseases, previous eye surgery, age under 18 or over 40 years old, pregnant women and eyes with AL < 21.00 mm or AL > 27.50 mm.

The present study adhered to the ethical principles of the Declaration of Helsinki. All the participants were carefully informed about the purpose of the study and written informed consent was acquired from each subject. Institutional Review Board (IRB) approval was also obtained from the ComEtico Campania Sud (CECS), prot. n°16544.2.2

### 2.2. Clinical and Instrumental Examination

All patients underwent a complete eye examination consisting of the following:

- slit lamp inspection;
- visual acuity test;
- fundus examination.

All recruited patients had a best corrected visual acuity >20/25 and absence of abnormalities at both slit lamp and fundus examinations. The evaluation also included a spectral domain optical coherence tomography (OCT) evaluation with enhanced depth image (EDI) mode (Spectralis; Heidelberg Engineering; Heidelberg, Germany, version 6.0) and AL measurement with IOLMaster (Carl Zeiss Meditec AG, Jena, Germany, version 5.4.4.0006). The OCT exams were performed without the instillation of mydriatics eye drops, given the related ChT thinning in the temporal sector [23].

#### 2.2.1. OCT Analysis

OCT scans were performed with the Heidelberg Spectralis spectral domain (SD) device. The SD-OCT uses a spectrometer to detect the interference pattern of the light reflected from different tissue layers [27]. SD-OCTs can produce high-speed images of the tissue with high axial resolution, for this reason, they are widely used in ophthalmology to perform the diagnosis and management of several pathologies [27]. The OCT analysis was performed using the OCT software measurement tool (Heidelberg Eye Explorer HEYEX, version 5.3; Heidelberg Engineering). The macular region was evaluated through a horizontal 30° linear OCT B-scan passing through the fovea, with an average of 100 frames for every B-scan. Only scans through the fovea and images with a high signal-to-noise ratio (minimum of 20 dB) were included and used for the ChT evaluation. All subjects were analyzed between 2:00 p.m. and 4:00 p.m. in order to reduce bias related to the diurnal ChT variations.

The ChT assessment was performed at the subfoveal level, at 1500 µm nasally and temporally from the fovea, perpendicularly from the hyper-reflective line of the outer border of the retinal pigment epithelium to the hyper-reflective sclero-choroidal junction centered at the base of the fovea.

#### 2.2.2. AL Measurements

The AL measurements were performed in the routinely used method with an IOL Master 500, which is a GRI-based biometer; it measures AL utilizing partial coherence interferometry (PCI) technology. AL was measured with a semiconductor laser diode that emits a dual beam of infrared light (780 nm). The signal produced by the interference between the light reflected from the tear film and the one reflected by the retinal pigment epithelium is received by the photodetector, which is used to calculate the optical distance between the corneal surface and the retina. This optical distance is used to calculate the geometrical intraocular distances [28]. In addition, IOLMaster 500 measures keratometry values at a 2.5 mm zone on the anterior cornea with six measured points, and it measures anterior chamber depth (ACD) using lateral slit illumination [28]. The AL measurements were taken utilizing the phakic option available on the biometer. The AL values were then corrected utilizing the following formula from De Bernardo et al.:

ALc = −0.017 + 0.996 * AL

where ALc is the corrected AL, and AL is the AL detected with the optical biometer by utilizing the phakic option [18]. Contrary to many other AL adjustments, ALc should be used independently from a specific AL range, because it is based on the assumption of a systematic error correction in the optical biometer, noting the influence of lens opacity and GRI on the reliability of the AL measurement. For this reason, the AL of all participants was corrected [29].

### 2.3. Statistical Analysis

Data collection was performed with Microsoft Excel and statistical analysis with IBM SPSS software (International Business Machine Corporation, Armonk, NY, USA, version 26.0). The Kolmogorov–Smirnov Test was performed to assess the normal distribution of the data. The Student T-test was used for pair-wise comparisons of AL and ALc. The correlations between ChT with AL and ALc were investigated using the Pearson correlation coefficient (r) and the Pearson coefficient of determination. Pearson’s analysis with bootstrap was also performed in additional evaluation according to eye-side subgroups. In all analyses, *p* values < 0.05 were considered statistically significant. Additionally, the mean, standard deviation, median, minimum, and maximum were calculated for each data point.

The preliminary sample size was calculated with G*Power software (Version 3.1.9.7, Faul, Erdfelder, Lang, & Buchner, 2020.Available at https://www.gpower.hhu.de, access date: 10 October 2024). Given a conventional effect size q of 0.6, it was estimated that a sample size of 47 eyes for each group (94 eyes in total) would be necessary, with a significance level of 5% and a test power of 80%.

## 3. Results

In total, 100 eyes of 50 healthy subjects (23 males and 22 females) with a mean age of 27.6 ± 2.3 years (ranging from 24 to 35 years) were evaluated. Both AL measurements and ChT data were normally distributed (all *p* > 0.050). The descriptive statistics of the data are reported in Table 1.

A statistically significant decrease was found with ALc compared to AL: mean difference was −0.11 mm ± 0.06 mm (*p* < 0.001).

**Figure 1:**
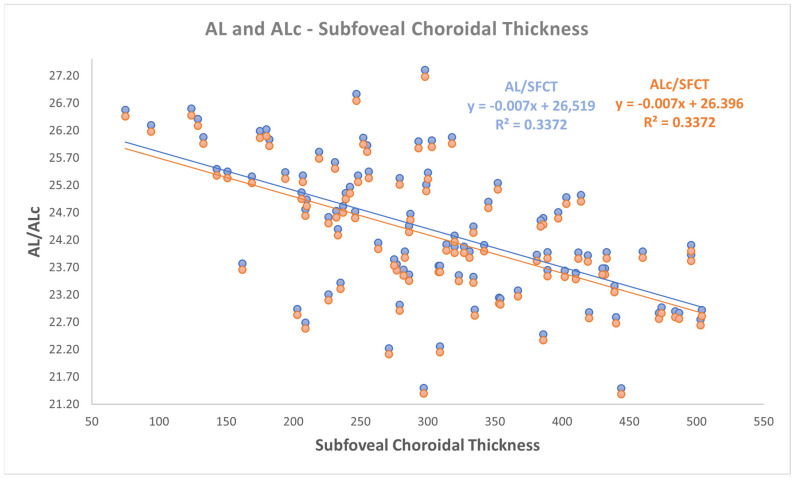
The relationship between both axial length (AL) and corrected AL (ALc) with subfoveal choroidal thickness.

**Figure 2:**
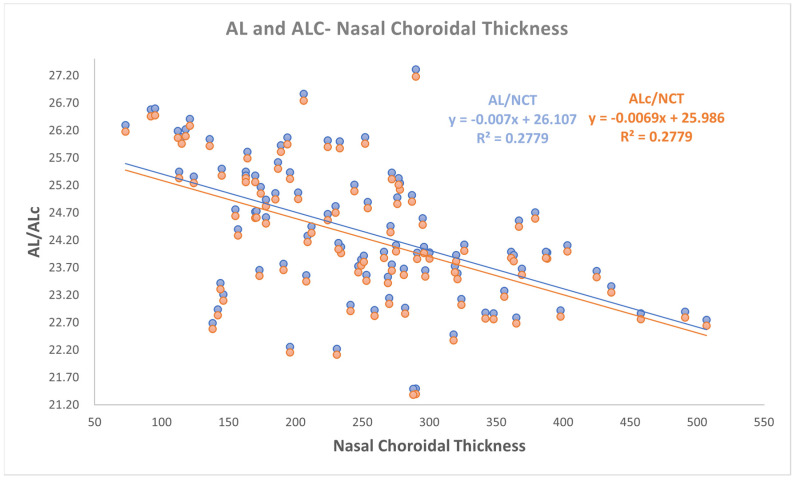
The relationship between both axial length (AL) and corrected AL (ALc) with nasal choroidal thickness.

**Figure 3:**
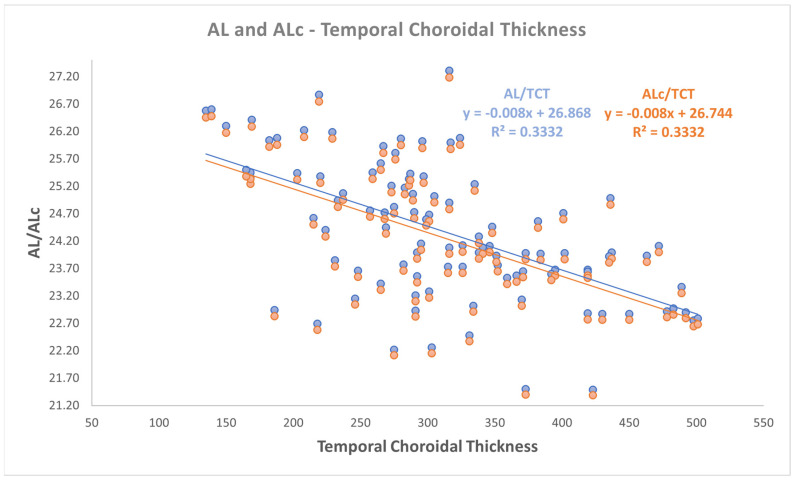
The relationship between both axial length (AL) and corrected AL (ALc) with temporal choroidal thickness.

Even if a small change in slopes and intercepts in linear relationships analysis was found, as reported in Figure 1, Figure 2 and Figure 3, a moderate negative correlation persists between both AL and ALc and ChT in different sectors. In detail, the findings were as follows:

- a negative correlation was found between both AL and ALc with subfoveal ChT (r: −0.581, *p* < 0.001);
- a negative correlation was found between both AL and ALc with nasal ChT (r: −0.527, *p* < 0.001);
- a negative correlation was found between both AL and ALc with temporal ChT (r: −0.577, *p* < 0.001).

Correlation analyses between AL and the choroidal parameters also with coefficient of determination according to Pearson are defined in Table 2 and in Figure 1, Figure 2 and Figure 3.

Also, subgroups according to eye sides were normally distributed (*p* > 0.050) and the additional evaluation according to eye side subgroups is reported in Table 3.

## 4. Discussion

In the literature, a negative correlation between ChT and AL has been clearly described.

Bulut A et al. studied 53 myopic children’s eyes, for SE up to −0.75 diopter, and compared them to 64 emmetropic ones, with SE between −0.50 and +0.50 D. Myopic eyes showed a significantly thinner choroid than the emmetropic control eyes in subfoveal site and in three additional sites carried out every 750 μm temporal (T1, T2 and T3) and nasal (N1, N2 and N3) to the fovea. Furthermore, in the same population including myopic and emmetropic children, ChT was negatively correlated with AL and positively correlated with SE [7].

Gupta P et al. focused on the distribution of choroidal thickness in 448 myopic eyes compared to 116 emmetropic ones and concluded that highly myopic eyes have a significantly thinner peripapillary choroid and showed a different distribution of thickness compared with the controls [8].

Sonoda S et al. examined a large (7500 µm width) choroidal area of 180 right eyes of 180 healthy volunteers with an AL range between 21.9 and 27.7 mm, showing no statistically significant correlation between AL and total choroidal area, luminal area and stromal area. However, the authors found an inverse correlation between AL and the ratio of the luminal to stromal area, thus considering the luminal area to be more affected by reduction than the stromal one [9].

El-Shazly A et al. elaborated on the effect of ocular parameters, such as spherical equivalent (SE) and axial length (AL), on ChT. They assessed the ChT of patients with different degrees of myopia. AL showed a significant positive correlation with SE (r = 0.90 and *p* < 0.001) but a negative correlation with subfoveal, 2 mm nasal, temporal, upper and lower ChTs (r = −0.78, −0.83, −0.75, −0.76, and −0.69, respectively) (*p* < 0.001). Moreover, they checked if the ChT is affected uniformly throughout the macula or at different rates in different macular regions. In fact, they observed the thinnest choroid in the 2 mm nasal and the thickest choroid in the 2 mm temporal in mild and moderate myopia, in the 2 mm lower in the sever myopia, and in the 2 mm upper in advanced myopia. SE and AL were discovered to be the most important determinants for subfoveal nasal and temporal ChTs; the SE was the only determining factor for 2 mm upper ChT, while AL was the only determinant for 2 mm lower ChT [10].

Liu B et al. studied adult highly myopic eyes and confirmed AL as the most significant predictor of the sub foveal ChT [11].

All the above-mentioned studies analyzed studies according to AL groups or between specific AL cut-off values. However, these limits could be affected by the reliability of AL measurements. Firstly, authors evaluate AL correction related to IOL power calculation. In fact, to reduce the percentage of eyes with hyperopic refractive outcome, Wang et al. published several preoperative AL correction factors [19,20,21].

Cooke et al., with the so-called Cooke modified AL (CMAL), instead demonstrated that CMAL can simulate a sum-of-segments AL by using a biometer that works with GRI. In fact, it is well-known that GRI-based biometers give a shorter value of AL in short eyes and longer values of AL in long eyes [22].

Contrary to the Wang–Koch AL-correcting factors [19,20,21,22], both ALc and CMAL should be applied not only in long eyes, but in eyes of all AL ranges [18,22,29].

Therefore, ALc was proposed based on the influence of lens opacity and GRI on the reliability of the AL measurement: in this way, a correcting factor applied to preoperative AL could eliminate any systematic error biometry [18]. IOLMaster 500 measures AL by utilizing a GRI, meaning that it uses a mean refractive index to calculate AL [29], so it is not able to internally correct such a systematic error, because it is inherent to the instrument operating principle [18,28,29]. Similarly, ALc should be also applied for all phakic AL measurements for all other GRI-based biometers, independently from both AL acquisition technologies and specific AL ranges [29]. Hence, even if our study found the same relationship between AL with ChT and between ALc with ChT, the cut-off values should be changed with consideration of the reliability of AL. For example, it is known that “High myopia” is defined as the presence of myopic refractive error associated with AL ≥ 26.00 mm [30,31], but when performing studies that analyzed ChT in myopic eyes, the cut-off values should be arranged according to the corrected AL.

To sum up, several sources of error could affect the reliability of AL measurement:

- Inaccurate measurement technique: for example, contact ultrasound biometry is known to be unreliable;
- Principle of function of optical biometer: GRI-based biometers are more sensitive to AL measurement error, rather than sum-of-segments biometers;
- Eye length, with less reliable AL values with GRI-based biometers in case of long eyes.
- Lens opacity, that could affect reliability of all types of optical biometers, but especially GRI-based biometers.
- In all these cases, both clinicians and researchers should pay attention in both AL and ChT evaluation.

A bias in these types of studies can be represented by accommodation. In fact, in this scenario, changes in crystalline lens could contribute to AL overestimation [12]. Particularly, Woodman EC et al. observed both AL and ChT changes accompanying accommodation in myopes and emmetropes. At baseline, myopes had an average AL of 24.73 ± 1.04 mm (*n* = 33) and emmetropes of 23.37 ± 0.81 mm (*n* = 16), with a high significantly difference (*p*< 0.001) between them. They described choroidal thinning by 9 ± 18 µm in myopic subjects during accommodation and by 7 ± 22 µm in emmetropes ones, with a statistically significant difference between the two groups (*p* < 0.05). Changes in AL and ChT were shown to have a highly significant but weak negative association at analysis of covariance (*p* < 0.001, r^2^ = 0.077, slope b = −0.321) [13].

Kaple D et al. likewise compared AL and ChT changes during accommodation in young adult emmetropes and myopes, especially at peripheral sites. Upon accommodation, the AL changes maximally on-axis (41 ± 17 μm) and minimally at the 20° nasal site (26 ± 17 μm). On the other hand, the overall ChT decreased by 21 ± 7 μm (*F*1276 = 23.85, *p* < 0.001). ChT decreased more in myopes (23 ± 11 μm) than emmetropes (17 ± 8 μm) (*p* = 0.02). The greatest decrease of 30 ± 14 μm occurred on-axis and the lowest one of 14 ± 14 μm occurred at 30° temporally. ChT changes account for about 60% of changes arising for AL: the authors concluded that AL and ChT changes during accommodation are not significantly correlated with any position [14].

This phenomenon could be explained by the action of the ciliary muscle, choroidal non-vascular smooth muscle, and choroidal blood flow. The ciliary muscle is involved in the accommodation process and its contraction could also be transferred to the choroid, causing choroidal drag and scleral circumference reduction. Choroidal smooth muscle and blood flow could change in tone during accommodation, contributing to this event [15,16,17].

This knowledge highlights the cause and the possible management of eventual refractive errors occurring after measuring AL with optical biometry. It suggests measuring AL immediately following accommodation or during accommodation with an instrument that also provides measurements of lens thickness.

As a vascular tunic of the ocular globe, choroid is richly vascularized and provides the oxygen and nutrients required for the retinal pigment epithelium and of the outer retina. For the same reason, it can be reached by toxins, autoantibodies, and immunocomplexes, and so it can be involved in the pathogenesis of various ocular disorders. Investigating the eventual adaptations of the negative correlation between ChT and AL in various pathologic cohorts can reveal new features of their pathogenesis. For example, De Bernardo M et al. confirmed the existence of a significant correlation between AL and all the choroidal parameters in a celiac group, except for CVI, in a study comparing the subfoveal ChT of celiac patients with healthy controls [2].

It was already proven that ALc played a clinically relevant role in IOL power calculation [18,29], and AL correction is also essential in different fields of biometry. In fact, several reasons can clarify the importance of utilizing ALc in ChT studies:

(1) Ignoring AL correction could introduce bias to the ChT evaluation of patients grouped by AL. It results in an unreliable estimation of ChT of patients with macular edema and subretinal fluid [23] and in a difficult comparison between these patients and their healthy controls. Grading and staging different ocular and systemic pathologies through ChT could be an interesting clinical and therapeutic skill, so its trustworthy use is decisive.
(2) AL correction is essential for both patients with cataracts and subjects that underwent GRI-based biometry, as discussed in previous paragraphs.
(3) Studies concerning myopia progression and ChT [32,33] should be carried out according to ALc. The choice of AL cut-off values should be taken according to corrected AL values.
(4) Not only AL cut-off values, but also evaluation of both AL and ChT changes, should be analyzed according to ALc [14,24,25].
(5) In addition, relationships between ChT and retinal diseases [34] should be analyzed with ALc values.
(6) In conclusion, patients’ stratification according to AL should be performed with ALc in ChT studies [26].

It should be noted that ALc by De Bernardo et al. [18] was developed by noting an apparent reduction in AL in post cataract surgery eyes: on this basis, they verified that the refractive index of the human lens could change according to the cataract grade, making AL measurement in GRI-based biometers unreliable [18]. De Bernardo et al. demonstrated the presence of a systematic error in AL measurement in all cataractous eyes, despite the AL range [18]. For this reason, lens thickness was not considered by ALc: this parameter could be unreliable in case of cataract. ALc has more strength in eyes with cataract, therefore it should be considered, especially in case of examination of cataractous eyes. The OCT examinations were performed without any mydriatic eye drops. This should be considered a point of strength of this study. In fact, a significant thinning of the ChT in the temporal sector following the administration of tropicamide + phenylephrine has been demonstrated [23]. Such thinning was not observed in the subfoveal or nasal zone, probably because the presence of the optic nerve in the nasal choroidal side could influence the ChT, or due to an asymmetric choroidal watershed and choroidal vessels in the temporal choroid. For these reasons, the nasal and subfoveal ChT are not affected by mydriatics [23]. Avoiding pharmacological mydriasis has guaranteed the maximum reliability of ChT data, even in the temporal sector.

Since AL measurement with GRI-based biometry is inaccurate because they assume a global refractive index for different structures (cornea, aqueous, lens, vitreous), ChT measurements should not be influenced by GRI, but it was demonstrated that brightness of OCT images can influence the measurements of choroidal parameters. Therefore, ChT should not be corrected but OCT evaluation must be performed by using the same parameters [35]. Furthermore, OCT’s light source is also a specific wavelength of light, thus it is also affected by the choroidal refractive index; therefore caution should be taken, especially when comparing different OCT devices in evaluation of choroidal parameters.

This study is affected by some limitations. Firstly, we evaluated both eyes for each subject. In fact, ocular measurements are more alike between fellow eyes. This similarity could cause a compounding of data; therefore, analyzing fellow eyes cannot be treated as if they were independent [36]. To overcome this problem, appropriate statistical analyses could be performed, such as the bootstrap or generalized estimating equations [36]. If the correlation between the left and right eyes of each patient is not accounted for in the statistical analysis, errors may occur in the obtained results: in fact, ignoring inter-eye correlation can lead to smaller *p*-values when both eyes are in the same group [36]. Another solution could be represented by the choice of using only one eye for each subject [36]. For this reason, we performed an additional analysis with bootstrap by evaluating subgroups based on the eyes’ side (Table 3) and similarly a strong negative linear correlation was found both with AL and ALc compared to ChT (all r < −0.500, all *p* < 0.050).

In addition, the relationship between CMAL and ChT was not evaluated because the lens thickness (LT) measurement, required for CMAL [22], was not available with IOL Master 500. On the other hand, Wang–Koch adjustments were not analyzed because they were designed specifically to reduce hyperopic refractive error in long eyes [19,20,21].

A future prospective is represented by confirming the same relationship is also present in the case of “atypical” eyes: for example, it is well-known that refractive surgery causes a keratometry flattening, but also an AL, anterior chamber depth, and central corneal thickness (CCT) decrease [37,38]. Therefore, when the impact of corneal changes on IOL power calculation [37,38] and of CCT reduction on intraocular pressure measurement [39] are well-known, the impact of AL decrease on ChT evaluation is less well-known.

In conclusion, a statistically significant linear correlation persists between ALc and ChT, as in to the uncorrected AL. Due to systematic errors in GRI-based optical biometry, we recommend recruiting and stratifying population according to ALc, in order to apply the correct cut-off values in ChT studies. Therefore, both AL and ChT changes should be evaluated according to ALc. 

## Figures and Tables

**Table 1:** Descriptive statistics of uncorrected and corrected axial length and nasal, subfoveal, and temporal choroidal thickness.

Parameter	AL	ALc	ChT Nasal	ChT Subfoveal	ChT Temporal
**MEAN**	24.36 mm	24.25 mm	250.57 µm	307.18 µm	313.72 µm
**SD**	1.23 mm	1.22 mm	93.03 µm	101.66 µm	88.86 µm
**MEDIAN**	24.12 mm	24.00 mm	250.00 µm	299.50 µm	301.00 µm
**MIN**	21.49 mm	21.39 mm	73.00 µm	75.00 µm	135.00 µm
**MAX**	27.31 mm	27.18 mm	507.00 µm	504.00 µm	501.00 µm

SD: standard deviation; Min: minimum; Max: maximum; AL: axial length, ALc: corrected axial length according to De Bernardo et al.; ChT: choroidal thickness.

**Table 2:** Correlation between uncorrected and corrected axial length and choroidal parameters.

Correlation	r	95% CI	R^2^	*p*-Value
AL—Subfoveal ChT	−0.527	−0.669 −0.383	0.3372	0.000
AL—Nasal ChT	−0.581	−0.705 −0.449	0.2779	0.000
AL—Temporal ChT	−0.577	−0.700 −0.451	0.3332	0.000
ALc—Subfoveal ChT	−0.527	−0.669 −0.383	0.3372	0.000
ALc—Nasal ChT	−0.581	−0.705 −0.449	0.2779	0.000
ALc—Temporal ChT	−0.577	−0.700 −0.451	0.3332	0.000

AL: axial length, ALc: corrected axial length according to De Bernardo et al.; ChT: choroidal thickness; r: correlation coefficient according to Pearson correlation analysis; 95% CI: 95% confidence interval around r according to Pearson correlation analysis; R^2^: coefficient of determination according to Pearson correlation analysis, *p*-value: level of significance according to Pearson correlation analysis.

**Table 3:** Correlation between uncorrected and corrected axial length and choroidal parameters in right eye and left eye subgroups.

Correlation	r—RE (*p*-Value)	95% CI—RE	r—LE (*p*-Value)	95% CI—LE
**AL—Subfoveal ChT**	−0.548 (0.000)	−0.727 −0.352	−0.621 (0.000)	−0.774 −0.412
**AL—Nasal ChT**	−0.546 (0.000)	−0.719 −0.334	−0.507 (0.000)	−0.697 −0.285
**AL—Temporal ChT**	−0.533 (0.000)	−0.711 −0.312	−0.624 (0.000)	−0.782 −0.421
**Alc—Subfoveal ChT**	−0.548 (0.000)	−0.727 −0.352	−0.621 (0.000)	−0.774 −0.412
**ALc—Nasal ChT**	−0.546 (0.000)	−0.719 −0.334	−0.507 (0.000)	−0.697 −0.285
**Alc—Temporal ChT**	−0.533 (0.000)	−0.712 −0.312	−0.624 (0.000)	−0.782 −0.421

RE: right eyes; LE: left eyes; AL: axial length, ALc: corrected axial length according to De Bernardo et al.; ChT: choroidal thickness; r: correlation coefficient according to Pearson correlation analysis; 95% CI: 95% confidence interval around r according to Pearson correlation analysis; *p*-value: level of significance according to Pearson correlation analysis.

## Data Availability

The datasets generated and analyzed during the current study are available from the corresponding author upon reasonable request.
